# *In-Situ* Probing Plasmonic Energy Transfer in Cu(In, Ga)Se_2_ Solar Cells by Ultrabroadband Femtosecond Pump-Probe Spectroscopy

**DOI:** 10.1038/srep18354

**Published:** 2015-12-18

**Authors:** Shih-Chen Chen, Kaung-Hsiung Wu, Jia-Xing Li, Atsushi Yabushita, Shih-Han Tang, Chih Wei Luo, Jenh-Yih Juang, Hao-Chung Kuo, Yu-Lun Chueh

**Affiliations:** 1 Department of Electrophysics, National Chiao-Tung University, Hsinchu 30010, Taiwan; 2 Department of Photonics and Institute of Electro-Optical Engineering, National Chiao-Tung University, Hsinchu 30010, Taiwan; 3 Department of Materials Science and Engineering, National Tsing Hua University, Hsinchu 30013, Taiwan

## Abstract

In this work, we demonstrated a viable experimental scheme for *in-situ* probing the effects of Au nanoparticles (NPs) incorporation on plasmonic energy transfer in Cu(In, Ga)Se_2_ (CIGS) solar cells by elaborately analyzing the lifetimes and zero moment for hot carrier relaxation with ultrabroadband femtosecond pump-probe spectroscopy. The signals of enhanced photobleach (PB) and waned photoinduced absorption (PIA) attributable to surface plasmon resonance (SPR) of Au NPs were *in-situ* probed in transient differential absorption spectra. The results suggested that substantial carriers can be excited from ground state to lower excitation energy levels, which can reach thermalization much faster with the existence of SPR. Thus, direct electron transfer (DET) could be implemented to enhance the photocurrent of CIGS solar cells. Furthermore, based on the extracted hot carrier lifetimes, it was confirmed that the improved electrical transport might have been resulted primarily from the reduction in the surface recombination of photoinduced carriers through enhanced local electromagnetic field (LEMF). Finally, theoretical calculation for resonant energy transfer (RET)-induced enhancement in the probability of exciting electron-hole pairs was conducted and the results agreed well with the enhanced PB peak of transient differential absorption in plasmonic CIGS film. These results indicate that plasmonic energy transfer is a viable approach to boost high-efficiency CIGS solar cells.

Photovoltaics have been touted as a prominent candidate for solving energy crisis and greenhouse effect. Many efforts have been dedicated to enhance light absorption ability of photovoltaics by various light management engineering, including increase of effective optical path by scattering approach^1^, anti-reflection utilizing nanostructures^2^ and employing quantum dots as wavelength shifter^3^. However, the conversion efficiency of solar cell modules still remains insufficient to meet our demand and there is still a considerable gap to achieve desirable light-to-electricity conversion efficiency of these photovoltaics. In particular, even though the light absorption ability has been quite superior, the improvement in conversion efficiency is still hindered by the heat loss. Therefore, scientific consideration has emerged to increase the scope of boosting high-efficiency solar cells. Nowadays, several cutting-edge approaches, including the photovoltaics exploiting (1) intermediate-band^4^, (2) hot carrier collection^5^, and (3) multi-exciton generation etc^6^. have been proposed to reduce the heat loss and overcome the Shockley-Queisser limit in solar cells^7^.

CuIn_1−x_Ga_x_Se_2_ (CIGS) is one of the favorable materials for thin-film solar cells owing to the high environmental tolerance^8^, long-term stability^9^ and excellent absorption ability^10^. The highest record efficiency of 21.7% has been achieved by ZSW^11^, which is also the highest one in all thin-film photovoltaics to date. In the last decade, researchers have proposed a number of methods to improve the performance of CIGS-based solar cells, including the gallium-doping for bandgap engineering^12^, nanostructures for enhancing absorption^13^, alkali-doping for passivation^14^ and plasmonic effect for light confinement^15^. Among them, plasmonics is a state-of-the-art approach for improving the performance of various optoelectronic devices. The extraordinary optical properties originated from the collective oscillation of the triggered surface charges in nanostructures, also known as surface plasmon resonance (SPR), has been shown to substantially enhance the photon-electron coupling and applied to the optoelectronic devices such as laser^16^, light-emitting diodes^17^, photovoltaics^18^, and photodetectors^19^. Many researches have demonstrated that SPR could serve as effective schemes for boosting the conversion efficiency of solar cells by increasing the optical paths and promoting the absorption of incident light resulted from the enlarged scattering cross-section and amplified near-field^7^,^20^,^21^,^22^. In addition, plasmonic energy transfer is another important subject attracting an ever increasing research interest and has been considered as a possible scheme to break the Shockley-Queisser limit by transferring the radiative and non-radiative energies to excite more electron-hole pairs^23^,^24^. Recently, the photocatalytic activity enhanced by plasmonic resonant energy transfer from Au to Cu_2_O has been verified by Wu *et al.*, confirming that plasmonic energy transfer can be a promising alternative to harvest the energy in addition to conventional electron–hole separation routes prevailing in semiconductor^23^,^25^.

Very recently, we had successfully demonstrated the merits of using plasmonic Au nanoparticles (Au-NPs) for the efficiency enhancement of CIGS thin-film flexible photovoltaics (TFPV)^15^. However, the mechanisms governing the plasmonic energy transfer in these systems remained unclear and, in this regard, further systematic investigations are needed. In this study, by elaborately analyzing the lifetimes and zero moment for hot carrier relaxation based on the data obtained by ultrabroadband femtosecond pump-probe spectroscopy, we demonstrated that the plasmonic energy transfer in Cu(In, Ga)Se_2_ solar cells incorporated with Au-NPs could be probed *in-situ*. In particular, the signals of the enhanced photobleach (PB) and waned photoinduced absorption (PIA) attributable to the SPR effect of Au-NPs could be probed *in-situ* by measuring the transient differential absorption (Δ*A*) spectra. The results revealed that substantial carriers can be excited from ground state to lower excitation energy levels, which can reach thermalization much faster with the aid of SPR effect. Thus, direct electron transfer (DET) was implemented to enhance the photocurrent of CIGS solar cells. Furthermore, based on the extracted hot carrier lifetimes, it was confirmed that the improved electrical transport might have been resulted primarily from the reduction in the surface recombination of photoinduced carriers through enhanced local electromagnetic field (LEMF). Finally, theoretical calculation for resonant energy transfer (RET)-induced enhancement in the probability of exciting electron-hole pairs was conducted and the results agreed well with the enhanced PB peak of transient differential absorption in plasmonic CIGS film.

## Experimental

CIGS thin films investigated in this study were prepared by pulsed laser deposition (PLD)^26^. The KrF excimer laser with the wavelength of 248 nm was employed as the laser source with the following operation conditions: pulse duration = 20 ns, pulse energy = 400 mJ, and pulse repetition rate = 10 Hz. The CIGS thin films were deposited on soda-lime glass (SLG) substrates for transmittance measurements. The thin film deposition was carried out in a vacuum chamber and the background pressure was kept at 4 × 10^−6^ Torr. The target to substrate distance was fixed at 4 cm. The substrate temperature was monitored by a thermocouple attached to the substrate holder and was kept at an optimal temperature of 500 °C during the deposition processes. The Au-NPs with diameter of ~10 nm were randomly dispersed on the CIGS film, resulting in a large variation in the SPR position and local electromagnetic field distribution.

**Figure 1:**
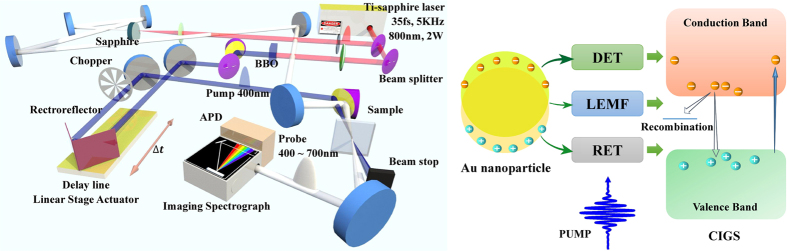
(**a**) The schematic diagrams of a non-collinear optical parametric amplifier (NOPA) and a 400 nm pump-ultrabroadband probe system. (**b**) The schematic presentation of various plasmonic energy transfer processes between Au-NPs and CIGS in plasmonic CIGS thin film. Also shown in the diagram are the excitation and recombination paths in CIGS.

For ultrabroadband pump-probe spectroscopy measurements, a non-collinear optical parametric amplifier (NOPA) for generating visible laser pulses was used, whose spectral width is adequately broad to sustain sub-10 fs visible pulses as shown in Fig. 1(a). A regenerative chirped pulse amplifier (Legend USP-HE; Coherent) seeded with a Ti:sapphire laser oscillator (Micra 10; Coherent) was introduced to pump and seed the NOPA. The amplifier generates 35-fs pulses with a central wavelength of 800 nm, a repetition rate of 5 kHz and an average power of 2 W. The laser beam from the regenerative amplifier is separated into two beams by a beam splitter. One beam is used to generate a second harmonic of 400 nm *via* BaBiO_3_ crystal to pump the samples. The other beam is focused on a sapphire plate to induce self-phase modulation in order to generate a broad visible spectrum, extending from 470 to 700 nm with an approximately constant phase. A mechanical delay stage was employed to vary the time delay between the pump and probe pulses. The intensities of these beams are adjusted *via* a variable neutral density filter and the ratio of the pump to the probe intensities is set to be about ten for the weakest excitation. The probe pulse is dispersed by a polychromator (SP2300i; Princeton Instruments) into a 196-branch fiber bundle, which is connected to avalanche photodiodes (APDs). Finally, the time-resolved transient differential signals at 196 probe wavelengths are simultaneously detected at the APDs. The signals detected at the APDs are sent to a multichannel lock-in amplifier to acquire a signal with a high signal-to-noise ratio.

## Results and Discussion

**Figure 2:**
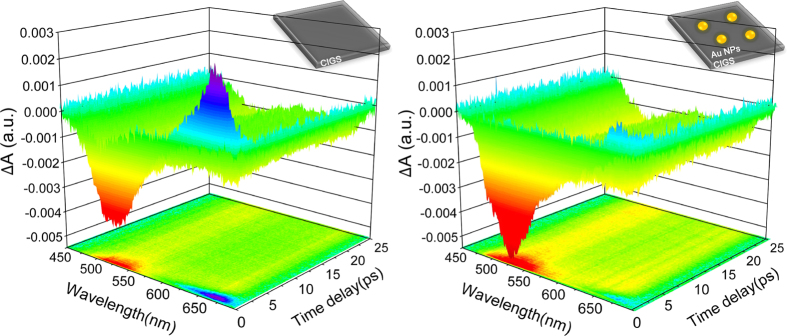
Three-dimensional plots of transient differential absorption Δ*A* spectra at different probing wavelengths and delay times in (**a**) pristine CIGS and (**b**) plasmonic CIGS thin films.

It is well known that femtosecond pump-probe spectroscopy is a powerful tool for investigating ultrafast carrier dynamics^27^,^28^,^29^,^30^,^31^. The non-equilibrium carriers can be immediately generated while the pumping pulses excite the semiconductors and their ultrafast relaxation processes reflected from the transient differential absorption signals Δ*A* were recorded by each probing pulse (see Fig. 1(b)). The measurement results of Δ*A(ω,t)* at different probing wavelengths for pristine CIGS (without Au-NPs) and plasmonic CIGS (with Au-NPs) thin films obtained by the present ultrabroadband pump-probe system are shown in Fig. 2(a,b), respectively. The negative part of Δ*A* in both spectra is generally attributed to the photobleach (PB) mechanism associated with the reduction of carriers in ground state. The results indicate that the carriers were effectively excited from the ground state by the pump beam, in some cases even reaching to the saturated state. In contrast, the positive part of Δ*A* at longer wavelengths is attributed to photoinduced absorption (PIA), which arose from the further excitation of excited carriers to higher energy levels^24^. The result indicates that photoexcited carriers in the plasmonic CIGS thin films were significantly increased and quenched in lower excited states with the incorporation of Au NPs.

**Figure 3:**
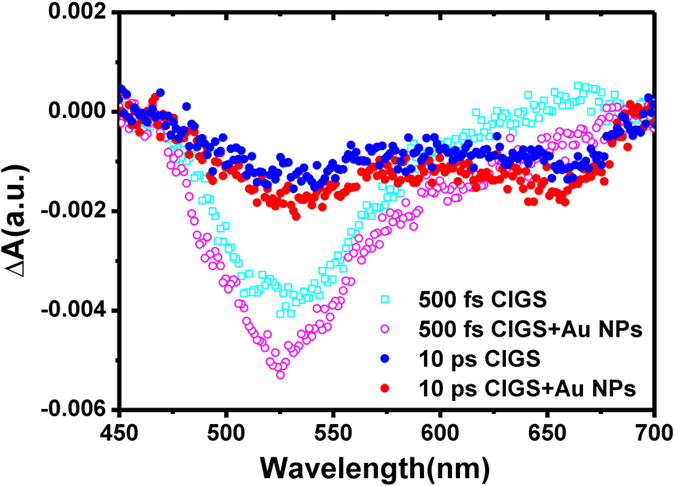


In order to elucidate the evolution of photoinduced species and SPR in detail, we extracted the Δ*A* signals at delay times of 500 fs and 10 ps for the pristine CIGS and plasmonic CIGS samples as shown in Fig. 3. At 500 fs, the negative change of Δ*A* assigned to PB was the dominant signal observed in both samples at shorter wavelengths of 450~600 nm. It is because that in this regime much more carriers are excited from the ground state and reaches the saturated state in probing spectrum. At longer wavelengths (>600 nm), the positive change in Δ*A*, corresponding to PIA signal, indicates that the excited carriers were re-excited to the higher energy levels, also known as free carrier absorption. Note that, with the existence of Au-NPs, the PB signal at 500 fs in plasmonic CIGS thin films is significantly enhanced whereas the PIA signal is substantially waned, suggesting that the SPR effect from Au-NPs might play a prominent role here. The SPR of Au-NPs can be triggered by incident pulses and instantaneously generates a strong near-field in the vicinity^15^. Nevertheless, the response of such strong near-field is diminishing quickly in short timescale through the dephasing process of surface plasmon oscillation^24^. As a result, with the existence of Au-NPs, more excited carriers are being generated by the strong near-field because of the larger light-harvesting via SPR at early time. However, owing to the degrading coherency of SPR, the effectiveness of carrier excitation decreases quickly and the signals of Δ*A* appear to almost identical at a time scale of ~10 ps. On the other hand, SPR also obviously suppresses the PIA mechanism, implying that the heat loss from hot-carrier relaxation process can be effectively reduced by the incorporation of Au-NPs. Moreover, at longer wavelengths, an evolution of PIA signal to PB signal featuring the process of hot-carrier population relaxation within few picoseconds was recorded, which undoubtedly lend strong support to our interpretation on the abovementioned photoinduced species^24^.

**Figure 4:**
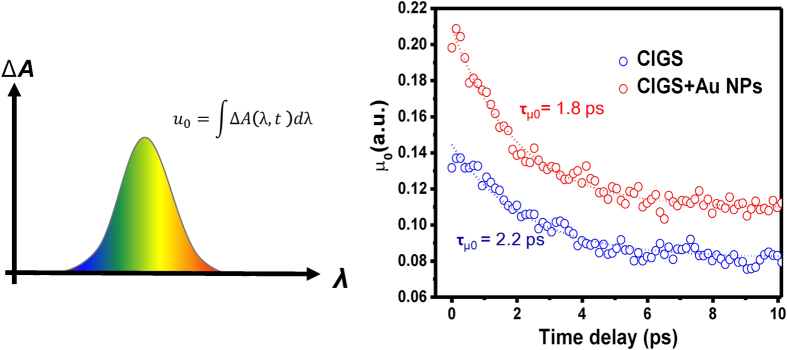
(**a**) An illustration of zero moment (μ_0_) analysis. (**b**) Evolution of zero moment for pristine CIGS and plasmonic CIGS thin films.

**Figure 5:**
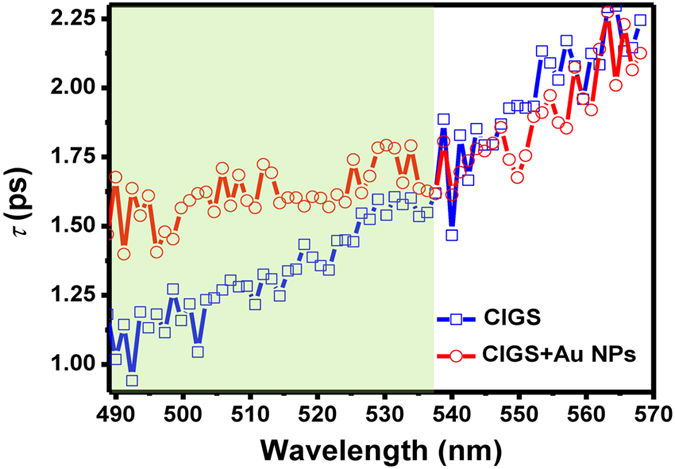
The wavelength-dependent lifetimes extracted from Δ*A* within 490–570 nm in Fig. 2.

Moment analysis has been developed as a statistical method for describing the population evolution of the excited carriers^32^. Figure 4(a) shows the calculation results of zero-order integral of the differential absorption to wavelengths, which is defined as zero moment (μ_0_). The magnitude of zero moment represents the amount of excited carriers and its decay time constant (τ_μ0_) features the evolution in carrier population. Figure 4(b) displays the calculated results of the zero moment for the pristine CIGS and plasmonic CIGS films in PB dominant region. It is apparent that, at delay time *t* = 0, the amplitude of μ_0_ increases by more than 50% and τ_μ0_ also becomes faster when Au-NPs are incorporated into the CIGS films, suggesting that more carriers are excited from the ground state by plasmonic effect. The result could also qualitatively explain the observed suppression of the PIA mechanism in plasmonic CIGS thin films. Because of faster thermalization (shorter τ_μ0_), the re-excitation to higher energy level for the excited carriers becomes scarcer. It is noted that, alternatively, the faster evolution in carrier population could also be attributed the leakage path to Au-NPs. In any case, according to the thermodynamic analysis within Shockley-Queisser model^7^, minimization of losses during carrier thermalization is always preferred for obtaining high-efficiency solar cells. Therefore, the present results imply that the plasmonic effect is a viable approach to substantially decrease the heat losses in CIGS solar cells and, thus, is potentially advantageous for hot carrier solar cells. Figure 5 shows the decay time constant (τ_recom_) extracted from each Δ*A* in the PB dominant region from Fig. 2, with which the time constants can be associated with the lifetime of photoinduced carriers and photocurrent extraction in solar cells. Note that many studies have suggested that the photoinduced carriers inevitably suffer defective traps in the polycrystalline CIGS film, particularly, nearby the surface of the CIGS thin film where the stacking of atoms were dramatically terminated^33^,^34^. A gradual decrease in lifetime with shorter probing wavelengths was observed in Fig. 5, presumably due to the fact that the shorter wavelengths mostly probed the photoinduced carrier recombination process close to the surface. Our previous study revealed that the open-circuit voltage (V_oc_) and filling factor (FF) of CIGS TFPV were significantly improved with the incorporation of Au-NPs, which may be related to the reduction of surface recombination by the SPR-induced strong near-field proximity to Au-NPs^15^. Figure 5 also shows the extended carrier lifetimes in shorter probing wavelengths, which is attributed to the existence of the strong near-field. The phenomenon of lifetime extension is expected to weaken with the longer wavelength due to attenuated SPR field in CIGS, which is indeed well corresponding to the trend observed in Fig. 5.

Our previous study and the abovementioned experimental results have suggested that the SPR effect resulted from Au-NPs does offer a unique opportunity for developing high efficiency CIGS solar cells, by substantially concentrating the incident photon energy into plasmonic oscillations and increasing light absorption of active region by the enlarged scattering cross-section at a near infrared region. The enhanced local electromagnetic field (LEMF) induced by SPR also played an important role as light harvesting antenna in visible light range, indicating that utilization of Au-NPs is capable of improving the light absorption ability of the CIGS film throughout the visible to near-infrared spectral ranges. Furthermore, suppression of surface recombination by LEMF was found to significantly improve the photocurrent extraction. However, in addition to these effects, other processes of plasmonic energy transferring have been proposed and developed. For instance, direct electron transfer (DET) and resonant energy transfer (RET) process were proposed to be the two primary routes for transferring energy from metallic nanoparticles to the semiconductor to excite electron-hole pairs in the semiconductors^23^. In essence, DET occurs after initial excitation and subsequent decoherence of the SPR, thus depends mainly on the alignment between the Fermi level of the plasmonic metal and band levels of the semiconductor, which in turn allows the population of hot carriers transferring from the plasmonic metal to the semiconductor^35^,^36^,^37^. In the present case, DET mechanism could contribute, at least partially, to the enhancement of population of excited carriers in the CIGS thin films (Fig. 4b). However, exact quantitative analysis on this effect needs further specifically designed experimental characterizations, such as employment of insulating interlayer between the plasmonic metal and semiconductor to prevent hot carriers transfer directly. In any case, our results of zero moment analysis have validated that the SPR effect does accelerate population evolution process of hot carriers and reduce heat loss, implying that DET could be an additional channel to effectively enhance the photocurrent of CIGS solar cell.

**Figure 6:**
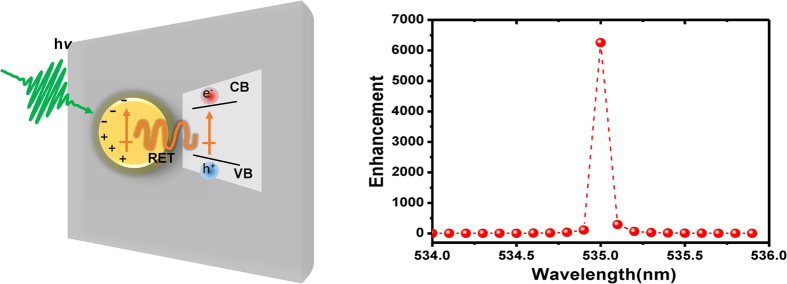
(**a**) A schematic representation of resonant energy transfer (RET) mechanism. CB: conduction band. VB: valence band. (**b**) The calculated RET-induced enhancement of carrier transition rate by Eq. (1).

Very recently, the RET mechanism has been considered as a new route of the SPR energy transfer. This relatively new frontier concept suggests that the electromagnetic field can be the medium for transferring the plasmonic energy in a non-radiative manner^23^. Comparing to the enhanced interband carrier transition rates of carriers in the semiconductor *via* LEMF, the proposed RET process represents another approach to induce charge separation in semiconductors *via* a non-radiative mechanism. As depicted schematically in Fig. 6(a), in this scenario, the electron-hole pairs in the semiconductor can be excited in a non-radiative manner by the relaxation of localized surface plasmon dipole interaction. Plasmon-induced RET in the near-field is analogous to Förster resonance energy transfer (FRET)^38^, wherein the near-field of SPR replaces the fluorescent system in FRET. In order to gain some insight of whether or not RET mechanism is prevailing in the present Au-NPs incorporated CIGS thin films, theoretical calculations were performed. In this model, the SPR of Au-NPs triggered by the incident electromagnetic wave and the collective oscillation of charges were treated in the form of dipole moment behavior. The diploe moment of the SPR can be approximately calculated from the Mie theory as 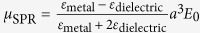, where *ε* is the dielectric constant, *a* is the radius of Au-NPs, and *E*_0_ is the electric field of the incident EM wave, respectively^23^. The enhancement of the carrier transition probability arising from the non-radiative RET interaction of *μ*_SPR_ and the semiconductor can be determined by quantum electrodynamics (QED) theory and expressed as^23^:


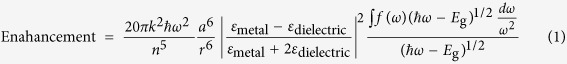


where *n* is the refractive index, *κ* is an orientation factor, *r* is the distance between the two dipoles, *f* (*ω*) is the normalized Lorentzian function and *E*_g_ is the bandgap of the semiconductor, respectively. Figure 6(b) exhibits the calculated probability enhancement of exciting electron-hole pairs in CIGS by using Au-NPs with diameter of 10 nm. It exhibits that the enhancement peak locating at 535.5 nm is very close to the negative peak related to PB displayed in Fig. 2(b). The result evidently demonstrates that the RET mechanism might have indeed contributed to the observed extra electron-hole pair excitations in CIGS with the incorporation of Au-NPs *via* the SPR effect. These results suggest that proper manipulations in plasmonic energy transfer could be a viable approach for boosting the efficiency of CIGS solar cells.

## Conclusions

In summary, we have demonstrated that, by using the ultrabroadband femtosecond pump-probe spectroscopy and elaborately analyzing the lifetimes and zero moment for hot carrier relaxation, the distinct features of plasmonic energy transferring associated with the Au-NPs incorporated Cu(In,Ga)Se_2_ solar cells can be probed *in-situ*. The transient differential absorption spectra signals attributed to PB were enhanced by the SPR near the Au-NPs, whereas the same SPR effect appeared to suppress the signals attributable to PIA process. The results suggested that excitation of carriers from ground state to lower excitation energy levels was substantially enhanced due to the existence of SPR. The evolution of carrier population was also faster with existence of SPR, revealing the heat loss was effectively reduced by SPR effect induced by Au-NPs. Furthermore, improvements of the electrical transport by reducing the surface recombination via LEMF was confirmed though the analysis of extracted lifetime in different probing wavelengths. Finally, the theoretical calculations based on the resonance energy transfer-induced enhancement in probability of exciting electron-hole pairs gave excellent agreement with enhanced PB peak of transient differential absorption in plasmonic CIGS films, suggesting that by properly manipulating the plasmonic energy transfer with the incorporation of suitable metallic nanostructures could be a viable approach for boosting the efficiency of CIGS solar cells.

## Additional Information

**How to cite this article**: Chen, S.-C. *et al.* *In-Situ* Probing Plasmonic Energy Transfer in Cu(In, Ga)Se_2_ Solar Cells by Ultrabroadband Femtosecond Pump-Probe Spectroscopy. *Sci. Rep.* **5**, 18354; doi: 10.1038/srep18354 (2015).
